# A new model of preoperative systemic inflammatory markers predicting overall survival of osteosarcoma: a multicenter retrospective study

**DOI:** 10.1186/s12885-022-10477-8

**Published:** 2022-12-30

**Authors:** Xianying Huang, Yongjin Liu, Weifeng Liang, Kai Luo, Yiwu Qin, Feicui Li, Tianyu Xie, Haibiao Qin, Juliang He, Qingjun Wei

**Affiliations:** 1 Department of Trauma Orthopedic and Hand Surgery, First Affiliated Hospital of Guangxi Medical University, Nanning, China; 2 Department of Spinal Surgery, the First Affiliated Hospital of Guangxi University of Chinese Medicine, Nanning, China; 3 Clinical Laboratory of Guilin Hospital of Traditional Chinese Medicine, Guilin, China; 4 Department of Spinal Bone Disease, First Affiliated Hospital of Guangxi Medical University, Nanning, China; 5 Department of Bone and Soft Tissue Surgery, Guangxi Medical University Cancer Hospital, Nanning, China

**Keywords:** C-reactive protein-to-albumin ratio, Neutrophil-to-lymphocyte ratio, Nomogram, Osteosarcoma

## Abstract

**Background:**

The purpose of this study was to investigate the significance of preoperative C-reactive protein-to-albumin ratio (CAR), neutrophil-to-lymphocyte ratio (NLR) and platelet-to-lymphocyte ratio (PLR) in predicting overall survival (OS) of osteosarcoma, to establish a nomogram of an individualized prognostic prediction model for osteosarcoma.

**Methods:**

Two hundred thirty-five patients with osteosarcoma from multiple centers were included in this study. Receiver operating characteristic (ROC) and Youden index were used to determine the optimal cutoff values ​​for CAR, NLR, and PLR. Univariate analysis using COX proportional hazards model to identify factors associated with OS in osteosarcoma, and multivariate analysis of these factors to identify independent prognostic factors. R software (4.1.3-win) rms package was used to build a nomogram, and the concordance index (C-index) and calibration curve were used to assess model accuracy and discriminability.

**Results:**

Univariate analysis revealed that the OS of osteosarcoma is significantly correlated (P < 0.05) with CAR, NLR, PLR, Enneking stage, tumor size, age, neoadjuvant chemotherapy (NACT), and high alkaline phosphatase. Multivariate analysis confirmed that CAR, NLR, Enneking stage, NACT and tumor size are independent prognostic factors for OS of osteosarcoma. The calibration curve shows that the nomogram constructed from these factors has acceptable consistency and calibration capability.

**Conclusion:**

Preoperative CAR and NLR were independent predictors of osteosarcoma prognosis, and the combination of nomogram model can realize individualized prognosis prediction and guide medical practice.

## Introduction

Osteosarcoma is the most common bone malignancy, which usually occurs in adolescents. It has a high degree of malignancy and rapid progression, often with early hematogenous metastasis and poor prognosis [1]. The traditional treatment methods are mainly surgery and postoperative chemotherapy, and the 5-year overall survival (OS) is less than 50% [2]. Since the 1970s, with the application of neoadjuvant chemotherapy(NACT), the five-year survival rate of non-metastatic osteosarcoma patients has risen to 60-70% [3]. However, the OS of patients with metastatic osteosarcoma is still less than 30% [4], and one of the important reasons may be that individualized prediction cannot be made in the early stage, so that more individualized treatment plans can be adopted to improve the prognosis.

At present, many factors have been reported to be related to the prognosis of osteosarcoma, including some clinical factors, imaging indicators, serology and molecular markers [5–8]. Clinicopathological features such as age, tumor location, tumor size, tumor stage, and pathological fracture have been confirmed to be correlated with the prognosis of osteosarcoma [9–12]. Serological and molecular markers such as alkaline phosphatase, p53, vascular endothelial Progeny factors have also been found to affect the prognosis of osteosarcoma [13–15]. In recent years, some inflammatory factors and nutritional factors, such as C-reactive protein-to-albumin ratio (CAR), platelet-to-lymphocyte ratio (PLR), neutrophil-to-lymphocyte ratio (NLR), etc., have also been reported to be related to the prognosis of osteosarcoma, and some are even considered to be Independent predictors of prognosis in osteosarcoma [16, 17]. However, at present, there is no unified view on the strength of each factor in predicting the prognosis of osteosarcoma at home and abroad, and the predictive effect of a single prognostic factor is limited. The nomogram can integrate multiple predictors, which has a greater advantage in the prediction of prognosis.

The nomogram was first introduced into oncology research in 1998 by Kattan et al. [18] to predict recurrence after radical prostatectomy. The nomogram can integrate multiple prognostic factors to achieve individualized prediction, and the nomogram is intuitive and easy to operate by clinicians and patients, so it has greater value in clinical practice. More and more literature reports confirm that the nomogram has more advantages in predicting the prognosis of various tumors, including breast cancer, rectal cancer, cholangiocarcinoma, soft tissue sarcoma, etc. [19–22]. In recent years, reports on the application of nomogram to predict the prognosis of osteosarcoma have gradually increased [10–12]. However, there are few reports on the use of inflammatory factors to construct nomograms, and even fewer reports on the use of CAR for individualized model prognosis prediction of osteosarcoma. Furthermore, the individualized prognostic model of osteosarcoma based on CAR and NLR combined clinicopathological factors has not been reported. Based on multi-center data, this study used CAR, NLR combined with clinicopathological indicators to construct an individualized prediction nomogram of osteosarcoma OS, and verified its effectiveness.

## Patients and methods

### Patients

Inclusion criteria: (1) In line with the postoperative pathological diagnosis of osteosarcoma; (2) The main treatment was performed in the First Affiliated Hospital of Guangxi Medical University, the Affiliated Cancer Hospital of Guangxi Medical University or the First Affiliated Hospital of Guangxi University of Traditional Chinese Medicine, especially the operation and postoperative Chemotherapy; (3) The follow-up time was more than 2 years. Exclusion criteria: (1) incomplete data; (2) surgery or postoperative chemotherapy in other hospitals; (3) follow-up time less than 2 years. A total of 453 patients with osteosarcoma from January 2012 to January 2020 were received treatment, and 235 patients met the criteria for this study. Institutional ethics committees approved this study.

### Prognostic factor

#### Clinicopathological factors

The following data of all patients were collected: age, sex, tumor site, location, Enneking stage, tumor subtype, pathological fracture, NACT, surgery. The tumor staging adopts the Enneking staging [23], the tumor types were divided into seven types including conventional osteosarcoma [24]. The NACT regimen is AP (cisplatin 75 ~ 100 mg/m^2^ + doxorubicin 45 mg/m^2^), MAP (cisplatin 75 ~ 100 mg/m^2^ + doxorubicin 45 mg/m^2^ + high-dose methotrexate 8 ~ 12 g/m^2^), completion of ≥ 3 cycles of chemotherapy before surgery was defined as completion of preoperative NACT. Standardized surgery requires extensive or radical surgical margins. After operation, AP (cisplatin 75–100 mg/m2 + doxorubicin 45 mg/m2) was continued. MAP (cisplatin 75–100 mg/m2 + doxorubicin 45 mg/m2 + high-dose methotrexate 8–12 g/m2) regimen was used for 10–12 cycles of chemotherapy.

### Hematology data and definitions

Hematologic parameters were collected within 1 week before diagnosis of osteosarcoma by biopsy. NLR is defined as neutrophil counts / lymphocyte counts; CAR is defined as C-reactive protein counts / albumin counts; PLR were defined as platelet counts / lymphocyte counts.

### Statistical analysis

SPSS 22.0 (SPSS, Inc., USA) statistical software package was used in our study. All categorical variables were used, and the χ2 test was used. We used Receiver operating characteristic (ROC) curve and Youden index to find the optimal cut-off values of CAR, PLR and NLR. OS was defined as the time from diagnosis to death, those lost to follow-up or surviving were censored data. Kaplan-Meier curves were used to draw survival curves and Log-rank was used for statistical significance. Univariate analysis using COX proportional hazards model to identify factors associated with OS in osteosarcoma, and multivariate analysis was used to identify independent prognostic factors based on these factors. *P* < 0.05 was considered statistically significant.

R software (4.1.3-win) rms package was used to build a nomogram, and all the prognostic factors in multivariate analysis were introduced. The predictive performance was quantified by C-index, and the Bootstrap method was used to repeat sampling 1000 times to draw the plot comparing nomogram predictions with actual observed survival.

## Result

### Clinicopathological features of patients with osteosarcoma

The clinicopathological features of all patients were analyzed (Table 1). 156 (66.38%) were male and 79 (33.62%) were female of the 235 patients, and their median age was 18 years (6–78 years) at diagnosis. Among all patients, 152 (64.68%) were conventional osteosarcoma. According to Enneking staging, 31 cases (13.19%) of stage I, 119 cases (50.64%) of stage II, and 85 cases (36.17%) of stage III were diagnosed. 140 patients (59.57%) received preoperative NACT.

**Table 1:** Clinical characteristics of all patients based on CAR, PLR and NLR

Characteristics	Total	CAR			PLR			NLR		
	*n*=235	LCAR	HCAR	P	LPLR	HPLR	P	LNLR	HNLR	P
**Age** (years)										
≤18	125	77	45	0.074	65	60	0.867	61	64	**0.019**
>18	110	55	55		56	54		37	73	
**Sex**										
Male	156	83	73	0.198	79	77	0.715	61	95	0.256
Female	79	49	30		42	37		37	42	
**Location**										
Extremities	208	116	92	0.731	108	100	0.712	90	118	0.176
Non-extremities	27	16	11		13	14		8	19	
**Tumor size**										
≤8cm	115	79	36	**<0.001**	71	44	**0.002**	54	61	0.110
>8cm	120	53	67		50	70		44	76	
**Enneking**										
I	31	26	5	**0.002**	19	12	0.222	18	13	**0.015**
II	119	66	53		64	55		54	65	
III	85	40	45		38	47		26	59	
**Subtype**										
Conventional osteosarcoma	152	85	67	0.772	78	74	0.945	59	93	0.596
Telangiectatic osteosarcoma	15	10	5		6	9		5	10	
Small cell osteosarcoma	4	1	3		3	1		3	1	
Parosteal osteosarcoma	27	15	12		14	13		12	15	
Periosteal osteosarcoma	5	4	1		3	2		3	2	
High grade surface osteosarcoma	2	1	1		1	1		1	1	
Low-grade central osteosarcoma	30	16	14		16	14		15	15	
**Pathological fracture**										
No	211	120	91	0.520	111	100	0.310	86	125	0.384
Yes	24	12	12		10	14		12	12	
**NACT**										
No	95	51	44	0.527	47	48	0.611	34	61	0.130
Yes	140	81	59		74	66		64	76	
**Surgery**										
No	31	13	18	0.086	10	21	0.021	11	20	0.451
Yes	204	119	85		111	93		87	117	
**alkaline phosphatase**										
** Normal**	99	62	37	0.089	49	50	0.602	47	52	0.126
** Elevated**	136	70	66		72	64		51	85	

*CAR* C-reactive protein-to-albumin ratio, *NLR* neutrophil-to-lymphocyte ratio, *PLR* platelet-to-lymphocyte ratio, *NACT* neoadjuvant chemotherapy

### Grouping definitions for CAR, NLR and PLR

**Fig. 1:**
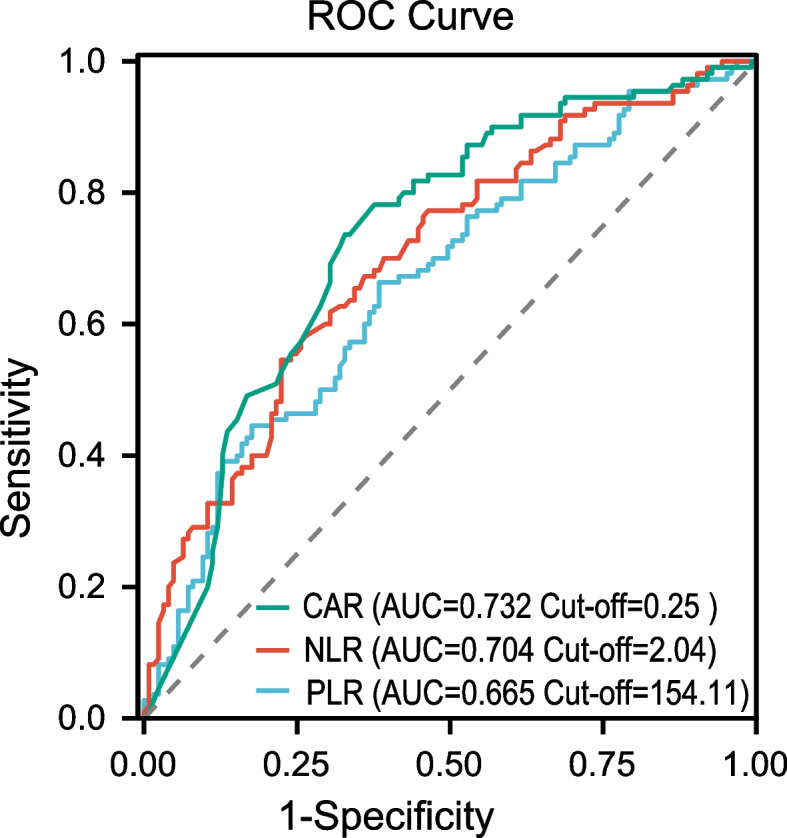
ROC curves and optimal cut-off of C-reactive protein-to-albumin ratio (CAR), neutrophil-to-lymphocyte ratio(NLR) and platelet-to-lymphocyte ratio (PLR). The areas under the curves for CAR, NLR and PLR were 0.733, 0.703 and 0.665, respectively

The best cut-off values ​​for these inflammatory factors to predict OS were (Fig. 1): CAR, 0.25 (AUC: 0.733); NLR, 2.04 (AUC: 0.703); PLR, 154.11 (AUC: 0.665), which basing on the ROC curve. According to the optimal cutoff value, we defined CAR as HCAR (> 0.25) and LCAR (≤ 0.25), NLR as HNLR (> 2.04) and LNLR (≤ 2.04), and PLR as HPLR (> 154.11) and LPLR (≤  154.11).

### Associations between the CAR, NLR, PLR and clinicopathological features

To explore the relationship between CAR, NLR, PLR and the clinicopathological characteristics of osteosarcoma, we performed a comparison between the high and low groups of the above indexes (Table 1). Our results found that HNLR patients were older than LNLR patients (*P* = 0.019). The tumor sizes in the HCAR group was larger than that in the LCAR group (*P* < 0.001), similar result was also found between the HPLR group and the LPLR group (*P* = 0.002). The higher the Enneking stage, the higher the probability of HCAR and HNLR (*P* = 0.002, 0.015, respectively).

### Prognostic factors for osteosarcoma

**Fig 2:**
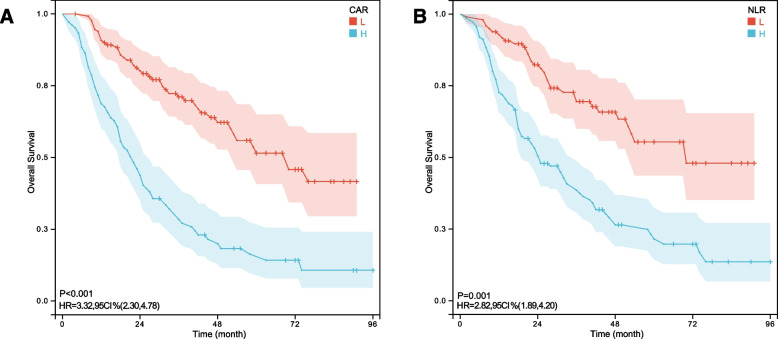
Overall survival Kaplan-Meier curves from 235 patients with osteosarcoma based on C-reactive protein-to-albumin ratio (CAR) (**A**) and neutrophil-to-lymphocyte ratio(NLR)(**B**)

The Kaplan Meier curve showed a lower 5-year OS of osteosarcoma in the HCAR group (*P* < 0.001) and the HNLR group (*P* = 0.001) (Fig. 2). Univariate analysis revealed that the OS of osteosarcoma is significantly correlated (*P* < 0.05) with CAR, NLR, PLR, Enneking stage, tumor size, age, neoadjuvant chemotherapy (NACT), and high alkaline phosphatase. Multivariate analysis confirmed that CAR, NLR, Enneking stage, NACT and tumor size are independent factors for OS of osteosarcoma (Table 2).

**Table 2:** Univariate and multivariate analyses for overall survival in all patients

Characteristics	Univariate			Multivariate		
	HR	95% CI	p	HR	95% CI	p
**Age** (years)						
≤18	1		**0.006**	1		0.587
>18	1.654	1.160-2.361		1.105	0.770-1.587	
**Sex**						
Male	1		0.281			
Female	0.809	0.551-1.189				
**location**						
Extremities	1		0.264			
Non-extremities	1.340	0.802-2.240				
**Tumor size**						
≤8cm	1		**<0.001**	1		**0.015**
>8cm	2.502	1.730-3.628		1.647	1.101-2.464	
**Enneking**						
I	1		**<0.001**	1		**<0.001**
II	5.164	2.073-12.864		3.206	1.249-8.229	
III	11.484	4.539-29.057		6.650	2.569-17.216	
**Subtype**						
Conventional osteosarcoma	1		0.612			
Telangiectatic osteosarcoma	1.619	0.781-3.356	0.196			
Small cell osteosarcoma	0.352	0.049-2.531	0.300			
Parosteal osteosarcoma	1.008	0.573-1.775	0.977			
Periosteal osteosarcoma	0.344	0.048-2.473	0.289			
High grade surface osteosarcoma	1.335	0.185-9.620	0.775			
Low-grade central osteosarcoma	0.851	0.475-1.526	0.589			
**Pathological fracture**						
No	1		0.905			
Yes	0.967	0.554-1.688				
**Surgery**						
No	1		0.302			
Yes	0.768	0.466-1.267				
**NACT**						
No	1		**0.001**	1		**0.017**
Yes	0.557	0.391-0.793		0.641	0.446-0.923	
**alkaline phosphatase**						
** Normal**	1		**0.016**	1		0.319
** Elevated**	1.574	1.089-0.273		1.216	0.828-1.786	
**CAR**						
LCAR	1			1		**<0.001**
HCAR	3.284	2.278-4.736	**<0.001**	2.265	1.535-3.343	
**PLR**						
LPLR	1		**0.001**	1		0.977
HPLR	1.825	1.272-2.619		1.006	0.675-1.498	
**NLR**						
LNLR	1		**<0.001**	1		**0.001**
HNLR	2.794	1.874-4.165		2.001	1.309-3.058	

*CAR* C-reactive protein-to-albumin ratio, *NLR* neutrophil-to-lymphocyte ratio, *PLR* platelet-to-lymphocyte ratio, *NACT* neoadjuvant chemotherapy

### Establishment and evaluation of Nomogram Prediction Model

**Fig. 3:**
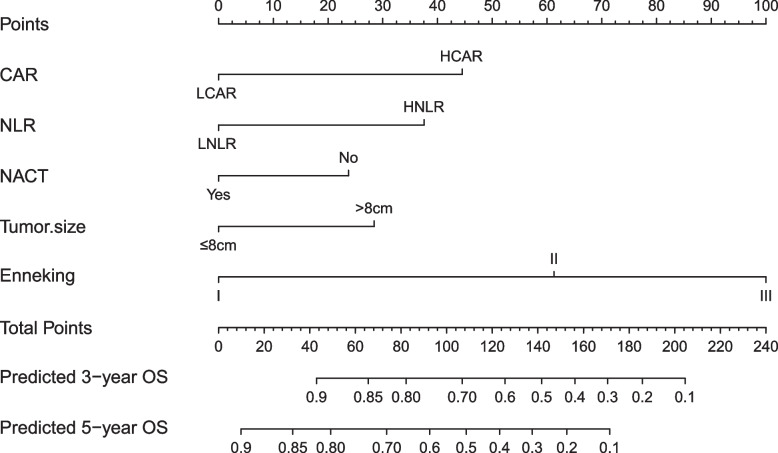
Nomogram with CAR, NLR and clinicopathological factors for predicting the probability Overall survival (OS) at 3- or 5-year

**Fig. 4:**
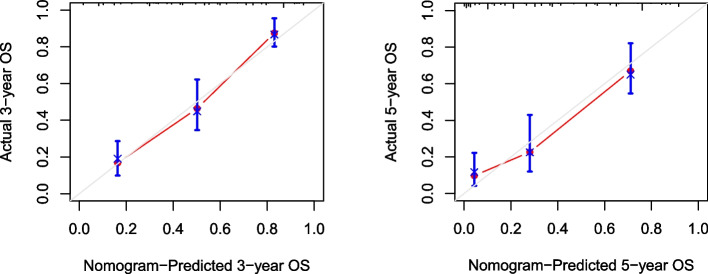
The calibration plot showed a high fit between actual and predicted 3- or 5-year Overall survival (OS)

Based on the multivariate analysis, the independent factors affecting OS were integrated, and R software was used establishing a nomogram for predicting OS in osteosarcoma (Fig. 3), the C-index of which was 0.781. A good agreement between nomogram predictions and actual observed 3-year and 5-year OS according to Calibration curves (Fig. 4).

## Discussion

This study is a multicenter study evaluating the association between OS with clinicopathological parameters and inflammatory biomarkers in osteosarcoma. Univariate analysis found that CAR, NLR, PLR, alkaline phosphatase, Enneking stage, age, tumor size, and neoadjuvant chemotherapy were associated with the OS of osteosarcoma. Further multivariate analysis found that CAR, NLR, Enneking stage, tumor size, and NACT are independent predictors of OS in osteosarcoma. Using the above clinically readily available factors, we established a visual nomogram model for the OS of osteosarcoma to achieve individualized prediction of osteosarcoma OS and provide an important tool for evaluating the prognosis of osteosarcoma.

From the first introduction of inflammatory factors to reflect the origin of tumors to the current in-depth exploration, it has been revealed that the inflammatory microenvironment has a significant impact on tumors [25–27]. At present, inflammatory response is considered to be an indispensable factor in tumor cell microenvironment involved in tumor proliferation, invasion, migration, metastasis, angiogenesis and tumor tissue damage repair [28–30], while malignant tumors in the process of malignant transformation, An inflammatory response is often stimulated, with increased peripheral blood neutrophils and decreased lymphocytes. As a major subpopulation of leukocytes, neutrophils can release nitric oxide or reactive oxygen species and remodel extracellular cells by producing proangiogenic growth factors and chemokines such as vascular endothelial growth factor matrix and PK2/Bv8, which in turn promotes tumor cell proliferation, metastasis, and angiogenesis [31]. Platelets, like neutrophils, can produce inflammatory cytokines and chemokines to participate in the inflammatory response and promote tumor angiogenesis, thereby promoting malignant progression [32]. However, lymphocytes play an important role in suppressing tumor progression because they can produce lymphokines, inhibit tumor cell proliferation and metastasis, and then cause tumor cytotoxic death [33]. Additionally, CRP is an acute-phase reactant that is regulated by proinflammatory cytokines, especially IL-6. The emergence of a systemic inflammatory state in cancer, reflected by elevated CRP levels, is often accompanied by a decrease in serum albumin concentration, sustained weight loss, impaired nutritional status, and increased mortality [34].

Inflammatory markers, such as CAR, PLR, NLR, etc., have been found to be related to the poor prognosis in some malignancies [35, 36]. However, there were few studies on the correlation between inflammatory factors and prognosis of osteosarcoma, especially the use of nomograms to predict the prognosis of osteosarcoma that made of inflammatory markers. Since CAR, NLR, and PLR are continuous variables, ROC curve was used in this study to determine their best cut-off values, and low and high groups were divided according to the value. Through univariate analysis, we found that there were significantly correlations between CAR, NLR, PLR and the OS of osteosarcoma. Interestingly, however, CAR and NLR were finally considered as independent predictors for patients with all types of osteosarcoma, while PLR ​​failed to show the ability to independently predict the OS of osteosarcoma, and these results were different from those of some previous studies [37], we believe that the reason may be that the inclusion criteria or indicators are inconsistent, such as the age composition of cases, tumor location and tumor stage. In order to further realize the individualized prognosis prediction of osteosarcoma, our study used CAR, NLR combined with other clinicopathological parameters to construct a nomogram with strong visualization, and the nomogram performed well in predicting the OS (C-index was 0.781). A good agreement between nomogram predictions and actual observed 3-year and 5-year OS according to Calibration curves, and it has a good clinical application value. As far as we know, reports using CAR and NLR were extremely rare to construct nomogram for osteosarcoma prognosis, and CAR and NLR can be obtained in routine peripheral blood detection. Therefore, CAR and NLR are expected to become biomarkers with good application value for evaluating the prognosis of patients with osteosarcoma.

## Limitation

Some limitations showed in this study. First, this is a retrospective study and may be subject to recall bias. Second, the number of study cases is small though it is a multi-center study, and the study results may have potential biases. A larger-scale prospective study is needed in the future to further verify the specific relationship between CAR, NLR and the OS of osteosarcoma. In addition, this study focuses on common clinical prognostic factors, and molecular markers that are not widely used in clinic, such as VEGF, p53, etc., have not been evaluated. Clinical factors combined with molecular markers to predict the OS of patients with osteosarcoma may have higher accuracy.

## Conclusion

In conclusion, CAR, NLR, Enneking stage, tumor size and NACT are independent predictors of OS in osteosarcoma. The nomogram established based on these prognostic factors is more intuitive to predict the OS of patients with osteosarcoma, and can realize the advantages of individualized prediction, and it is easy to obtain clinically, which is worth clinical promotion.

## Data Availability

All the data needed to achieve the conclusion are presented in the paper.

## Abbreviations

CAR: C-reactive protein-to-albumin ratio
NLR: neutrophil-to-lymphocyte ratio
PLR: platelet-to-lymphocyte ratio
OS: overall survival
ROC: Receiver operating characteristic
NACT: neoadjuvant chemotherapy.
